# Complex cells decrease errors for the Müller-Lyer illusion in a model of the visual ventral stream

**DOI:** 10.3389/fncom.2014.00112

**Published:** 2014-09-24

**Authors:** Astrid Zeman, Oliver Obst, Kevin R. Brooks

**Affiliations:** ^1^Department of Cognitive Science, ARC Centre of Excellence in Cognition and its Disorders (CCD), Macquarie UniversitySydney, NSW, Australia; ^2^Digital Productivity and Services Flagship (DPAS), Commonwealth Scientific and Industrial Research OrganisationMarsfield, NSW, Australia; ^3^Perception in Action Research Centre, Macquarie UniversitySydney, NSW, Australia; ^4^Department of Psychology, Macquarie UniversitySydney, NSW, Australia

**Keywords:** Müller-Lyer, illusion, HMAX, hierarchical, computational, model, visual, cortex

## Abstract

To improve robustness in object recognition, many artificial visual systems imitate the way in which the human visual cortex encodes object information as a hierarchical set of features. These systems are usually evaluated in terms of their ability to accurately categorize well-defined, unambiguous objects and scenes. In the real world, however, not all objects and scenes are presented clearly, with well-defined labels and interpretations. Visual illusions demonstrate a disparity between perception and objective reality, allowing psychophysicists to methodically manipulate stimuli and study our interpretation of the environment. One prominent effect, the Müller-Lyer illusion, is demonstrated when the perceived length of a line is contracted (or expanded) by the addition of arrowheads (or arrow-tails) to its ends. HMAX, a benchmark object recognition system, consistently produces a bias when classifying Müller-Lyer images. HMAX is a hierarchical, artificial neural network that imitates the “simple” and “complex” cell layers found in the visual ventral stream. In this study, we perform two experiments to explore the Müller-Lyer illusion in HMAX, asking: (1) How do simple vs. complex cell operations within HMAX affect illusory bias and precision? (2) How does varying the position of the figures in the input image affect classification using HMAX? In our first experiment, we assessed classification after traversing each layer of HMAX and found that in general, kernel operations performed by simple cells increase bias and uncertainty while max-pooling operations executed by complex cells decrease bias and uncertainty. In our second experiment, we increased variation in the positions of figures in the input images that reduced bias and uncertainty in HMAX. Our findings suggest that the Müller-Lyer illusion is exacerbated by the vulnerability of simple cell operations to positional fluctuations, but ameliorated by the robustness of complex cell responses to such variance.

## 1. Introduction

Much of what is known today about our visual perception has been discovered through visual illusions. Visual illusions allow us to study the difference between objective reality and our interpretation of the visual information that we receive. Recently it has been shown that computational vision models that imitate neural mechanisms found in the ventral visual stream can exhibit human-like illusory biases (Zeman et al., 2013). To the extent that the models are accurate reflections of human physiology, these results can be used to further elucidate some of the neural mechanisms behind particular illusions.

**Figure 1:**
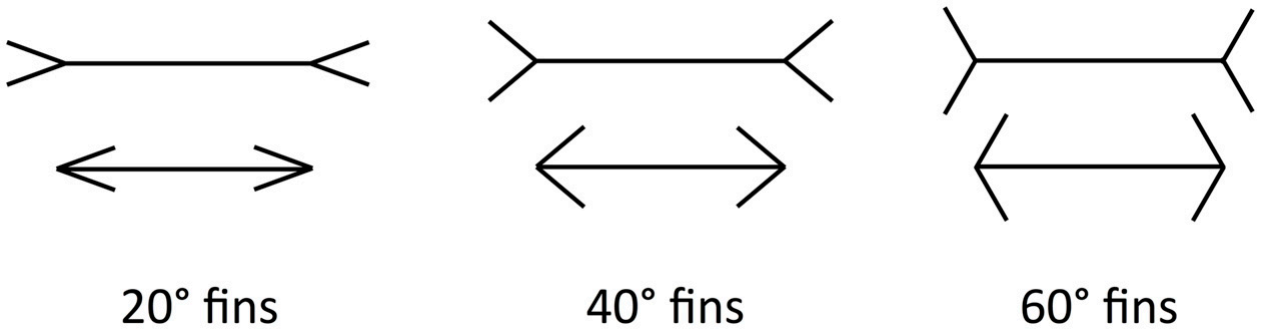
**The ML illusion in classical four-wing form**. Horizontal lines are the same length in all cases. The ML effect is stronger for more acute angles (Left) and weaker for more obtuse angles (Right).

In this paper, we focus on the Müller-Lyer Illusion (MLI), which is a geometrical size illusion where a line with arrowheads appears contracted and a line with arrow-tails appears elongated (Müller-Lyer, 1889) (see Figure 1). The strength of the illusion can be affected by the fin angle (Dewar, 1967), shaft length (Fellows, 1967; Brigell and Uhlarik, 1979), inspection time (Coren and Porac, 1984; Predebon, 1997), observer age (Restle and Decker, 1977), the distance between the fins and the shaft (Fellows, 1967) and many other factors. The illusion classically appears in a four-wing form but can also manifest with other shapes, such as circles or squares, replacing the fins at the shaft ends. Even with the shafts completely removed, the MLI is still evident.

Here, we employ an underused method to explore the Müller-Lyer illusion and its potential causes using an Artificial Neural Network (ANN). To date, few studies have used ANNs to explore visual illusions (Ogawa et al., 1999; Bertulis and Bulatov, 2001; Corney and Lotto, 2007). In some cases, these artificial neural networks were not built to emulate their biological counterparts, but rather to demonstrate statistical correlations in the input. One such example is the model used by Corney and Lotto (2007), consisting of only one hidden layer with four homogenous neurons, which few would consider to be even a crude representation of visual cortex. The work presented by Ogawa et al. (1999) used a network with three hidden layers of “orientational neurons,” “rotational neurons” and “line unifying neurons.” This network could roughly correspond to one layer of simple cells that provide orientation filters and one layer of complex cells that combine their output. However, this study presented no quantitative data and lacked a detailed description of the model, such as the size or connectivity of their network. Bertulis and Bulatov (2001) created a computer model to replicate the spatial filtering properties of simple cells and the combination of these units' outputs by complex cells in visual cortical area V1. Although they compared human and model data for the Müller-Lyer Illusion, their model centered only on the filtering properties of early visual neurons. These models do not adequately represent the multi-layered system that would best describe the relevant neural structures. Neuroimaging studies have shown areas V1, V2, V4, and IT are recruited when viewing the MLI (Weidner and Fink, 2007; Weidner et al., 2010) and hence the inclusion of operations from such visual ventral stream subdivisions is desirable. Therefore, studying the MLI in a computational model known to mimic these areas would provide a more biologically representative result.

In a previous report, we studied the MLI in a benchmark model of the ventral visual stream that imitates these cortical areas (Zeman et al., 2013). Following from our hypothesis that the MLI could occur in a model that imitates the structure and function of visual ventral areas, we demonstrated its manifestation in a biologically plausible artificial neural network. Although the models listed above are capable of reproducing the MLI, we believe our work provides a significant advance, being one of the first studies to model a visual illusion in a simulated replica of the ventral visual stream. In addition, our study contrasts with those above by employing techniques to train the model on multiple images before running a classification task and comparing the task of interest to a control. This allows us to separate the inner workings of the model from the input in the form of training images.

The model we recruit, HMAX (Serre et al., 2005), is a feed-forward, multi-layer, artificial neural network with layers corresponding to simple and complex cells found in visual cortex. Like visual cortex, the layers of HMAX alternate between simple and complex cells, creating a hierarchy of representations that correspond to increasing levels of abstraction as you traverse each layer. The simple and complex cells in the model are designed to match their physiological counterparts, as established by single cell recordings in visual cortex (Hubel and Wiesel, 1959). Here, we briefly describe single and complex cell functions and provide further detail on these later in Section 2.1. In short, simple cells extract low-level features, such as edges, an example of which would be Gabor filters that are often used to model V1 operations. The outputs of simple cells are pooled together by complex cells that extract combined or high-level features, such as lines of one particular orientation that cover a variety of positions within a visual field. Within HMAX, the max pooling function is used to imitate complex cell operations, giving the model its trademark name. In general, low-level features extracted by simple cells are shared across a variety of input images. High-level features are less common across image categories. The high-level features output by complex cells are more stable, invariant and robust to slight changes in the input.

HMAX has been extensively studied in its ability to match and predict physiological and psychological data (Serre and Poggio, 2010). Like many object recognition models, HMAX has been frequently tested using well-defined, unambiguous objects and scenes but has not been thoroughly assessed in its ability to handle visual illusions. Our previous demonstration of the MLI within HMAX showed not only a general illusory bias, but also a greater effect with more acute fin angles, corresponding to the pattern of errors shown by humans. Our replication of the MLI in this model allowed us to rule out some of the necessary causes for the illusion. There are a number of theories that attempt to explain the MLI (Gregory, 1963; Segall et al., 1966; Ginsburg, 1978; Coren and Porac, 1984; Müller-Lyer, 1896a,b; Bertulis and Bulatov, 2001; Howe and Purves, 2005; Brown and Friston, 2012) and here we discuss two. One common hypothesis is the “carpentered-world” theory—that images in our environment influence our perception of the MLI (Gregory, 1963; Segall et al., 1966). To interpret and maneuver within our visual environment, we apply a size-constancy scaling rule that allows us to infer the actual size of objects from the image that falls on our retina. While arrowhead images usually correspond to the near, exterior corners of cuboids, arrow-tail configurations are associated with more distant features, such as the right-angled corners of a room. If the expected distance of the features is used to scale our perception of size, when a line with arrowheads is compared to a line with arrow-tails that is physically equal in length, the more proximal arrowhead line is perceived as being smaller. Another common theory is based upon visual filtering mechanisms (Ginsburg, 1978). By applying a low spatial frequency filter to a Müller-Lyer image, the overall object (shaft plus fins) will appear elongated or contracted. Therefore, it could simply be a reliance on low spatial frequency information that causes the MLI. In our previous study, we were able to replicate the MLI in HMAX, allowing us to establish that exposure to 3-dimensional “carpentered world” scenes (Gregory, 1963) is not necessary to explain the MLI, as the model had no representation of distance and hence involved no size constancy scaling for depth. We also demonstrated that the illusion was not a result of reliance upon low spatial frequency filters, as information from a broad range of spatial frequency filters was used for classification.

In the current study, we set out to investigate the conditions under which the Müller-Lyer illusion manifests in HMAX and what factors influence the magnitude and precision of the effect. In particular, we address the following questions: (1) How do simple vs. complex cell operations within HMAX affect illusory bias and precision? (2) How would increasing the positional variance of the input affect classification in HMAX? Our principal motivation is to discover how HMAX processes Müller-Lyer images and transforms them layer to layer. Following from this, we aim to find ways to reduce errors associated with classifying Müller-Lyer images, leading to improvements in biologically inspired computational models. We are particularly interested in how hierarchical feature representation could potentially lead to improvements in the fidelity of visual perception both in terms of accuracy (bias) and precision (discrimination thresholds).

## 2. Materials and methods

### 2.1. Computational model : HMAX

To explore where and how the illusion manifests, we first examined the architecture of HMAX: a multi-layer, feed-forward, artificial neural network (Serre et al., 2005; Mutch and Lowe, 2008; Mutch et al., 2010). Input is fed into an image layer that forms a multi-scale representation of the original image. Processing then flows sequentially through four more stages, where alternate layers perform either template matching or max pooling (defined below). HMAX operations approximate the processing of neurons in cat striate cortex, as established by single cell recordings (Hubel and Wiesel, 1959). Simple cells are modeled using template matching, responding with higher intensity to specific stimuli, while complex cell properties are simulated using max pooling, where the maximum response is taken from a pool of cells that share common features, such as size or shape.

**Figure 2:**
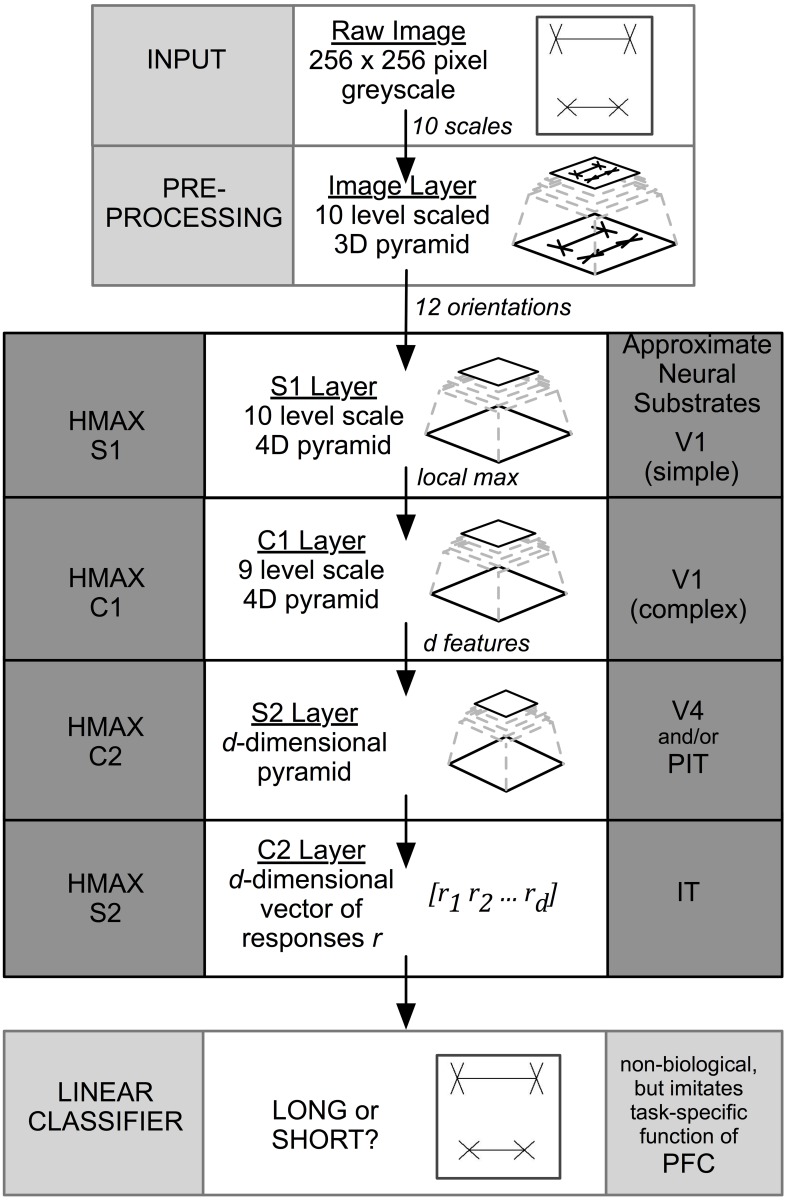
**HMAX architecture**. The input to the system is a 256 × 256 pixel image. The output is a binary classification. HMAX consists of alternating template matching (S layers) and feature pooling (C layers). The neural substrate approximations are taken from Serre et al. (2005).

Image information travels unidirectionally through four layers of alternating simple (“S”) and complex (“C”) layers of HMAX that are labeled S1, C1, S2, and C2. When the final C2 level is reached, output is compressed into a 1D vector representation that is sent to a linear classifier for final categorization. While previous versions of HMAX employed a support vector machine (SVM), in this paper we used the GPU-based version of HMAX (Mutch et al., 2010) that uses a linear classifier to perform final classification. The task for the classifier was to distinguish Long (i.e., top shaft longer) from Short (top shaft shorter) stimulus categories under a range of conditions, where the top or bottom line length varied by a known positive or negative extent. Figure 2 summarizes the layers and operations in the model. Precise details are included in the original papers (Serre et al., 2005; Mutch and Lowe, 2008; Mutch et al., 2010).

### 2.2. Stimuli: training and test sets(control and Müller-Lyer)

**Figure 3:**
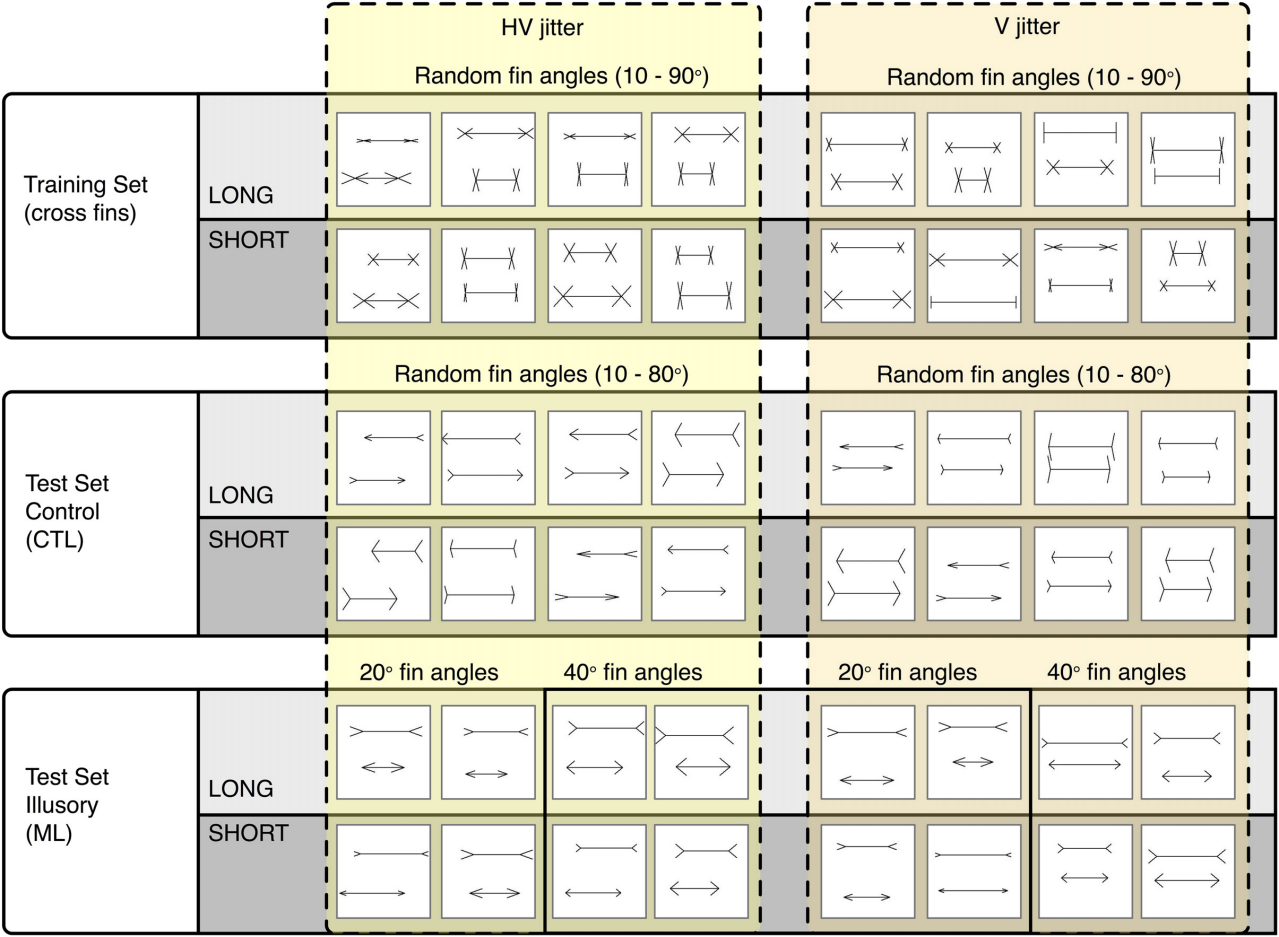
**Representative sample of images categorized as LONG or SHORT**. The Cross fin set (Top row) was used for training. The Control CTL set (Middle row) and Illusory ML set were both used for testing. Images are grouped into those that were jittered both horizontally and vertically (Left group) and those that were jittered only vertically (Right group).

To carry out our procedure, we generated three separate image sets: a training (cross fin) set, a control test set (CTL) and an illusion test set (ML). All images were 256 × 256 pixels in size, with black 2 × 2 pixel lines drawn onto a white background (see Figure 3). Each image contained two horizontal lines (“shafts”) with various fins appended. Each different image set was defined by the type of fins appended to the ends of the shafts. The fin type determines whether an illusory bias will be induced or not. Unlike the ML set, the cross fin and control test sets do not induce any illusions of line length in humans (Glazebrook et al., 2005; Zeman et al., 2013).

Within each two-line stimulus, the length of the top line was either “long” (L), or “short” (S), compared to the bottom line. The horizontal shaft length of the longest line was independently randomized between 120 and 240 pixels. The shorter line was varied by a negative extent randomly between 2 and 62 pixels for the training set, or by a known negative extent between 10 and 60 pixels for the test sets. The positions of each unified figure (shaft plus fins) were independently randomly jittered in the vertical direction between 0 and 30 pixels and in the horizontal direction between −30 and 30 pixels from center. The vertical position of the top line was randomized between 58 and 88 pixels from the top of the image while the bottom line's vertical position was randomized between 168 and 198 pixels. Top and bottom fin lengths randomized independently between 15 and 40 pixels. Fin lengths, line lengths and line positions remained consistent across all image sets. The parameters that varied between sets were fin angle, the direction of fins and the set size. If an image was generated that had any overlapping lines, for example, arrowheads touching or intersecting, these images were excluded from the sets.

Training images contained two horizontal lines with cross fins appended to the ends of the shafts (see Row 1, Figure 3). Fin angles were randomized independently for the top and bottom lines between 10 and 90°. Five hundred images per category (long and short) were used for training.

Two sets of test images were used, one as a control test set (CTL) and one as an illusion test set (ML). The CTL set used for parameterization contained left facing arrows for the top line and right facing arrows for the bottom line (see Row 2, Figure 3). CTL fin angles were randomized between 10 and 80° (the angles between top and bottom lines was the same). For parameterization, we used 200 images per category (totaling 400 images for both long and short) to test for overall accuracy levels with a randomized line length difference between 2 and 62 pixels. To establish performance levels for the control set, we tested 200 images per pixel condition for each category i.e., 200 images at 10, 20, 30, 40, 50, and 60 pixel increment differences for both short and long.

The ML set was used to infer performance levels for images known to induce an illusory bias in humans. In this ML set, all top lines contained arrow-tails and all bottom lines contained arrowheads (see Row 3, Figure 3). Fin angles for ML images were fixed at 20 and at 40° in two separate conditions. At the C2 layer, we tested 200 images for each pixel condition within each category (totaling 1200 images for the short category at 10, 20, 30, 40, 50, and 60 pixel length increments and 1200 for the long category). For all other layers (Input, S1, C1, and S2), we tested 100 images per pixel condition within each category. In each case we took the average of 10 runs, randomizing the order of training images. Classification results for the input, S1 and C1 levels are based on deterministic operations, without dependence on the weights developed during training. In these cases, randomizing the order of training images has no effect on classification results. To produce variation for these conditions, we generated additional test images that were randomized within the parameters specified above (with identical position ranges, fin angles, fin lengths, etc).

### 2.3. Procedure: learning, parameterization, illusion classification

Our method, established in Zeman et al. (2013), was carried out in three stages:

1. Training. Given a set of training images, a fixed-size network adjusted its internal weights to find the most informative features using unsupervised learning.
2. Test Phase 1: Parameterization. Using the CTL set, we ensured that the classifier was able to distinguish long from short images at an acceptable level of classification performance (above 85% correct), before testing with illusory stimuli. If performance fell below this level, we increased the size of the network and retrained (step 1).
3. Test Phase 2: Illusion classification. Using the ML set, we established the discrimination thresholds and the magnitude of the illusion that manifested in the model.

## 3. Results

### 3.1. Experiment I: classification of ML images after each level of HMAX

The aim of this experiment was to assess how simple and complex cell operations contribute toward bringing about the MLI. To this end, we examined the inner workings of HMAX, looking at classification performance for illusory images at each level of the architecture. We used a linear classifier to perform classification after each subsequent layer of HMAX, (which included processing of all previous layers required to reach that stage). Therefore, we ran classification on the Input only, on S1 (after information arrived from Input), on C1 (after information traversed through Input and S1 layers) and so on.

We first tested classification performance on our control images, which exceeded 85% when the size of the S2 layer was 1000 nodes. Using this network configuration, we tested classification on 20 and 40° ML images at the C2 level. We then tested classification at each layer of HMAX using the same illusory set.

**Figure 4:**
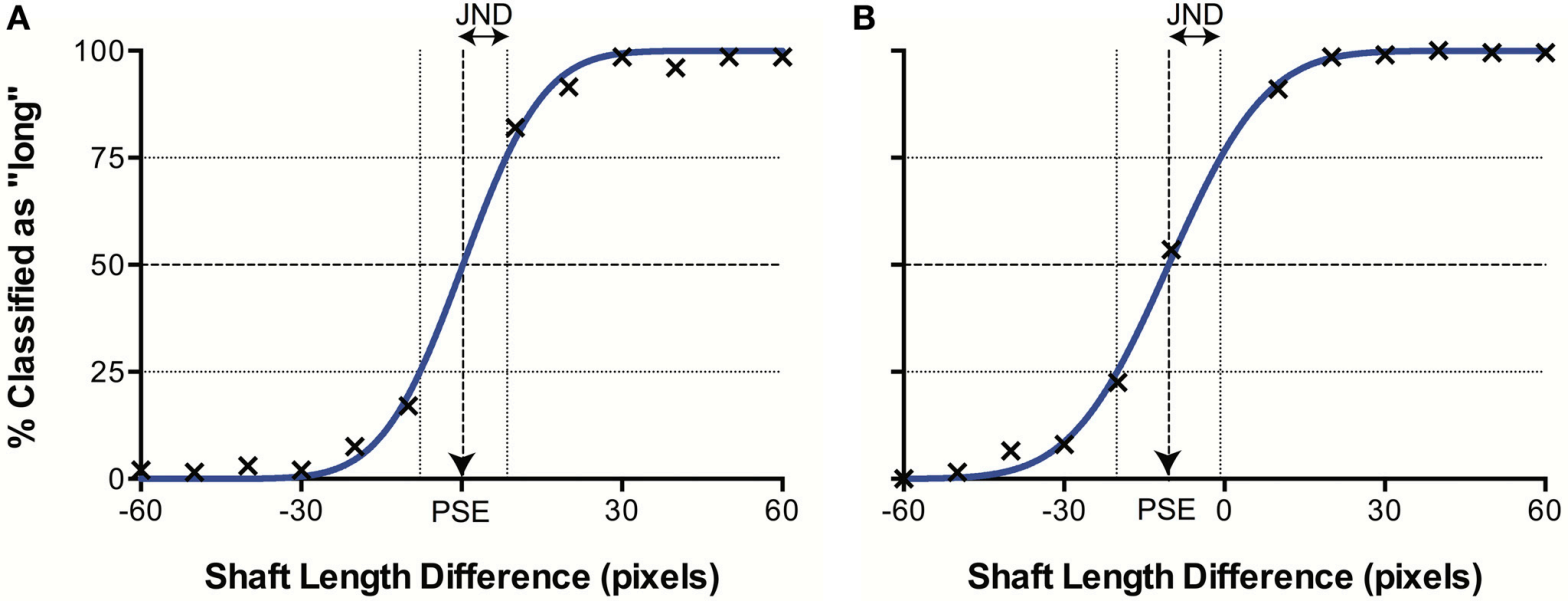
**Example data sets from **(A)** CTL and **(B)** ML (40°) conditions**. The best fitting curve (blue) allows derivation of the point of subjective equality (PSE) where classification is at 50%, and the just noticeable difference (JND), corresponding to the semi-interquartile difference.

When plotted in terms of the percentage of stimuli classified as “long” as a function of the difference in line length (top–bottom) for each separate data set (i.e., control images, illusory images with 20° fins and with 40° fins), we observed a sigmoidal psychometric function, characteristic of human performance in equivalent psychophysical tasks. The data were characterized by a cumulative Gaussian, with the parameters of the best-fitting function determined using a least-squares procedure. Figure 4 illustrates an example data set. When Gaussian curves did not fit significantly better than a horizontal line at 50% (chance responding) in an extra sum of squares *F*-test, the results were discarded (2 runs out of a total of 52). This allowed us to determine the Point of Subjective Equality (PSE) the line length difference for which stimuli were equally likely to be classified as long or short (50%), represented by the mean of the cumulative Gaussian. Here, PSEs are taken as a measure of accuracy, representing the magnitude of the Müller-Lyer Illusion manifested in the model. We also established the Just Noticeable Difference (JND) for each data set. The JND represents perceptual precision—the level of certainty of judgments for a stimulus type, and is indicated by the semi-interquartile difference of the Gaussian curve (the standard deviation multiplied by 0.6745). A higher JND represents greater uncertainty, and hence lower precision.

**Figure 5:**
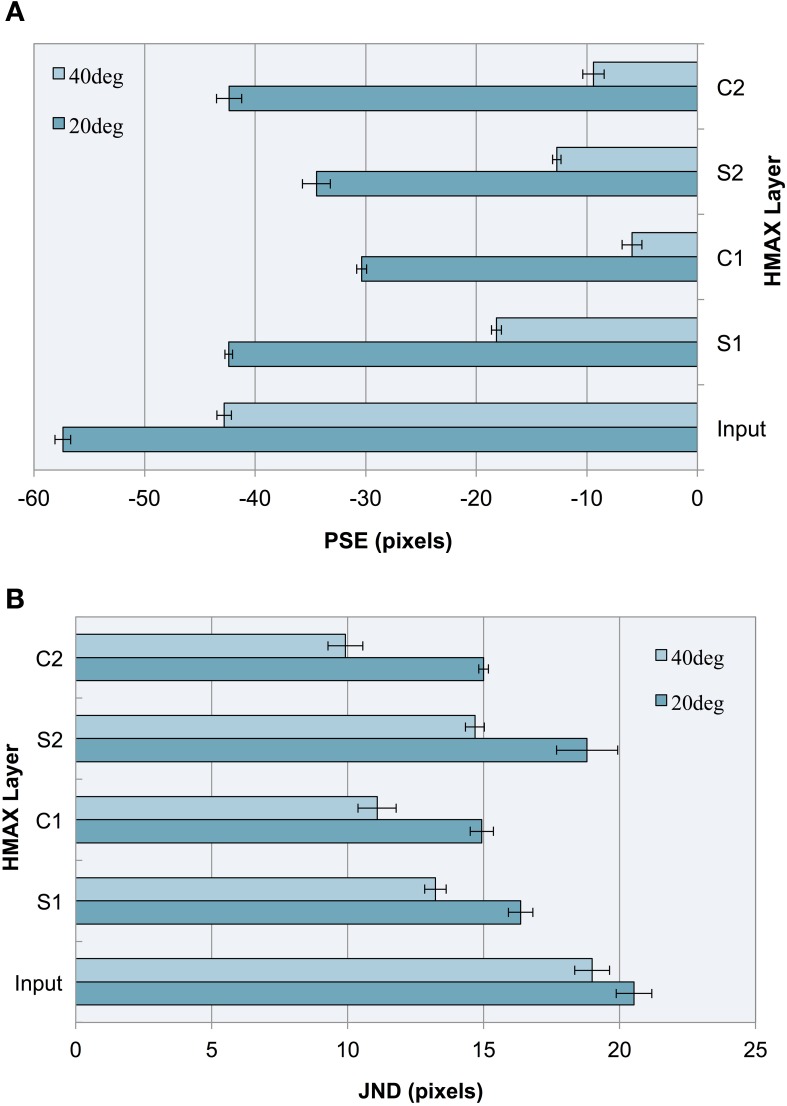
**Experiment I results as a function of the HMAX layer for images with 20° and 40° fins**. Error bars represent ± 1 s.e.m. across multiple runs. **(A)** Accuracy (PSEs). **(B)** Precision (JNDs).

As can be seen in our results (see Figure 5A), the model produces a pattern of PSEs for illusory images consistent with human bias. We see a larger bias for more acute angles (20°) vs. less acute angles (40°), a pattern that is also consistent with human perception. This constitutes a replication of our previous findings (Zeman et al., 2013) using a linear classifier, as opposed to a support vector machine (SVM), confirming that these findings are robust to the specific method of classification. These two trends are observable not only at the final C2 layer but at all levels of the architecture.

We observe that the illusion is present at the input level, suggesting that underlying statistical information may be present in our training images, despite careful design to remove bounding box cues and low spatial frequency information. The influence of image-source statistics on the Müller-Lyer illusion has already been studied using real-world environmental images and an input layer bias is to be expected (Howe and Purves, 2005). Because the aim of our study is to explore the Müller-Lyer within a biologically plausible model of the visual ventral stream, we are more interested in how the network would process the input. Our novel contribution, therefore, is to focus on how such information is transformed in terms of changes in accuracy and precision layer to layer as we traverse the cortical hierarchy within the HMAX network.

Observing the PSE for each HMAX layer after a linear classifier is applied, this experiment demonstrates three key findings:

1. Running a linear classifier on the raw images revealed a bias at the input level that would represent statistical influences such as those proposed by Howe and Purves (2005). However, each layer of the HMAX architecture counteracts this bias producing a reduction in PSE magnitude after every S and C layer is traversed, when compared to the input layer.
2. In the majority of cases (87.5% of the time), illusory bias and uncertainty is reduced after complex cell operations have been applied. A reduction in uncertainty and bias can be seen when comparing the PSE and JND for S1 vs. C1 layers, for both 20 and 40° fin angles in the illusion set. Going from S2 to C2, PSE is reduced for 40° angles but not for 20° angles in the ML set, whereas JND is reduced for all cases.
3. When simple cell operations follow complex, illusory bias and uncertainty is increased. At the S2 layer, we see an increase in PSE and in JND for both 20° and 40° ML images.

The observations concerning accuracy data are echoed for precision. In Figure 5B, we see a higher JND (lower precision) for images with more acute fin angles at all levels of HMAX architecture. Looking at each layer of the architecture, we see lower JNDs (higher precision) at each level of HMAX compared to the input alone. We also observe higher precision (smaller JNDs) following processing by complex cells, but lower precision when the output from these layers is passed through a simple cell layer. In the case of results for precision, these observations held without exception.

The contrast between results following processing by simple cell and complex cell layers encourages examination of the principal differences between the operations performed by these cells. The major distinction between S-layer and C-layer operations concerns the response to variance in the image. Unlike simple cells, whose outputs are susceptible to image variations such as fluctuations in the locations of features, complex cells' filtering properties allow them to respond similarly to stimuli despite considerable positional variance. When initially designing the training stimuli for HMAX, we wanted the system to build higher-level representations of short and long independent of line position, exact line length and of features appended to the shaft ends. This would require an engagement of complex cell functionality and less reliance on simple cell properties. To this end, we varied these parameters randomly in a controlled fashion to reduce reliance on trivial image details. If one of our training parameters were to be restricted, the architecture would be less able to build such robust concepts of short and long. Given that complex cells are designed to pool information across simple cells with similar response properties and fire regardless of small changes in the afferent information, decreasing the variance in one of our training parameters would underutilize C cell properties and the short and long concepts within HMAX would become less flexible. This is likely to reduce the overall categorization performance of the computational model. More specifically, we hypothesize that restricting positional jitter to only one dimension would decrease accuracy and precision with which HMAX categorizes Müller-Lyer images. If this hypothesis holds true, we would demonstrate that greater positional variance reduces illusory bias and uncertainty. To seek further support for this proposition, we remove horizontal positional jitter from all stimuli in our second experiment.

### 3.2. Experiment II: HMAX classification of ML images with reduced variance

In our previous experiment, we observed a reduction in the level of bias after complex cell operations and hypothesized that introducing greater variance in the input would further reduce bias levels. To test this, we measured classification performance for HMAX layer C2 under two conditions: (1) Using our default horizontal and vertical jitter (HV) and (2) Under conditions of decreased positional jitter (V). We reduced the positional jitter in our training and test images from two-dimensional jitter in both the horizontal and vertical dimensions to one-dimensional, vertical jitter. While the top and bottom lines and their attached fins in our training and test sets remained independently jittered vertically (between 0 and 60 pixels), we removed all horizontal jitter, instead centering each stimulus. The vertical position of the top line was randomized between 48 and 108 pixels from the top of the image while the bottom line's vertical position was randomized between 148 and 208 pixels. We thus maintained a maximal 60 pixel jitter difference per line while limiting jitter to only one dimension.

**Figure 6:**
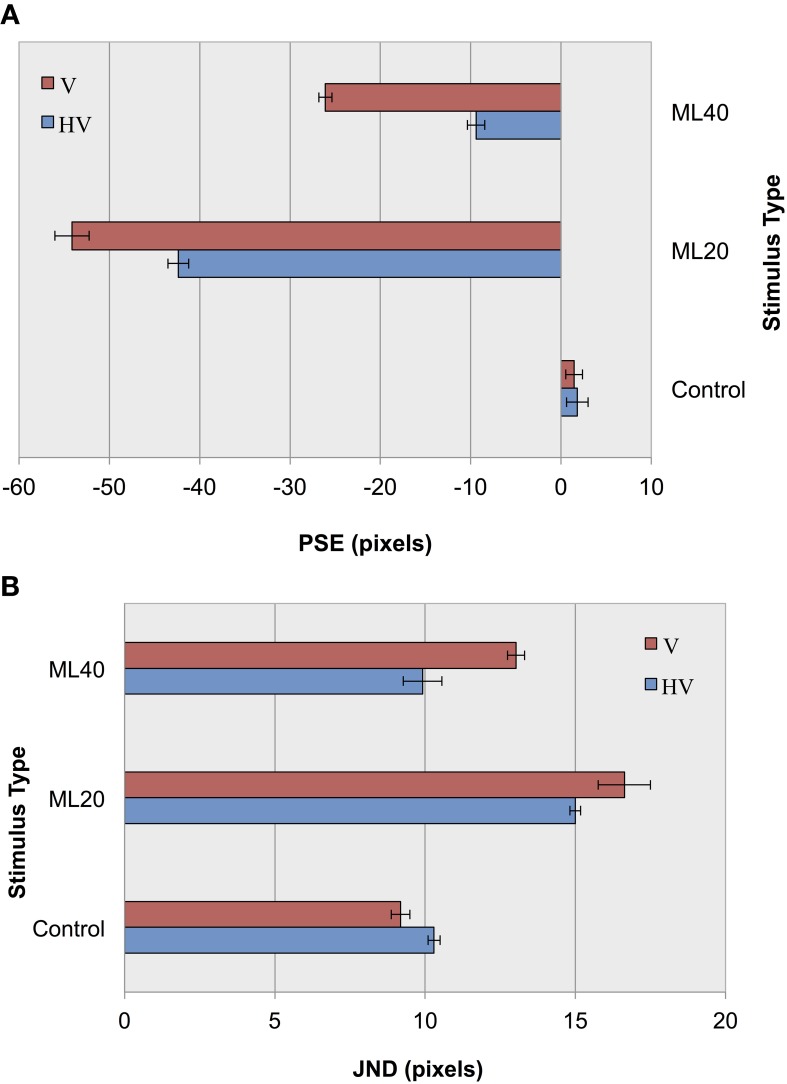
**Experiment II results as a function of jitter type for control images, and Müller-Lyer images with 20° and 40° fins. (A)** Accuracy (PSEs). **(B)** Precision (JNDs).

In an initial parameterization stage, we first tested performance using the CTL set, and found an overall classification score of 91.5% with an S2 size of 1000 nodes. The results of control and illusion image classification for our default jitter condition and for reduced positional jitter is shown in Figure 6. In terms of accuracy measurements (Figure 6A), it can be seen that for ML images PSEs are more extreme for V jitter only, compared to HV jitter. These results provide support for our hypothesis, demonstrating an increase in the magnitude of the Müller-Lyer effect for both 20 and 40° illusory conditions when reducing positional jitter, and hence image variance. As in before, the pattern of results for accuracy is echoed in terms of precision measurements (Figure 6B). Following the trend from our previous experiment, we see lower JND values for more obtuse angles compared to more acute angles. Comparing JND results for HV jitter with those for V jitter, we see that the classifier has higher precision when distinguishing short from long lines in the HV condition. In summary, decreasing the amount of positional variance in our stimuli increases bias and reduces the level of certainty in making decisions.

## 4. Discussion

Our aim for this study was to investigate the conditions under which the Müller-Lyer illusion manifests in HMAX and the factors that could influence the magnitude of the effect. Our primary motivation was to explore how hierarchical feature representation within HMAX affects classification performance. We ran two experiments performing binary image classification using HMAX. Images contained two horizontal lines that were jittered independently. Various configurations of fins were appended to the line shafts to create separate training and test images. Our first experiment compared the effects of operations performed by simple vs. complex cells by applying a linear classifier after each layer of HMAX when distinguishing long from short MLI images. Our second experiment examined HMAX classification of MLI images with decreased positional jitter.

The main finding from our first experiment is that the addition of any simple or complex cell layers reduces bias, compared to classification directly made on the input images. Illusory bias changes from layer to layer within a simple-complex cell architecture, with increases in MLI magnitude as information passes through simple layers. In most cases, the effect decreases as information passes through complex layers. The pattern of results for accuracy is replicated when measurements of precision are considered. All levels of HMAX show improved precision compared to classified input images, with further JND reductions caused by complex cell layers, and increases caused by simple cell layers. Proposing that the C layers' property of invariant responding may underlie their ability to increase accuracy and precision, we hypothesized that decreasing variance in the input images and re-training the network would increase the MLI. We chose to decrease the positional variance by removing horizontal jitter and including only vertical jitter for the stimuli in our second experiment. Consistent with our hypothesis, experiment 2 showed an increase in illusion magnitude for both 20 and 40° angles.

In this paper and in our previous study, we focused solely on the ML illusion in its classical four-wing form. It would also be possible to study other variants of the Müller-Lyer and other illusory figures to test more generally for the susceptibility of hierarchical artificial neural networks. Some variants of the Müller-Lyer to be tested could include changing the fins to circles (the “dumbbell” version) or ovals (the “spectacle” version) (Parker and Newbigging, 1963). Other monocular line length or distance judgment illusions occurring within the visual ventral stream may also manifest in similar hierarchical architectures, for example, the Oppel-Kundt illusion (Oppel, 1854/1855; Kundt, 1863).

Some illusions are moderated by the angle at which the stimulus is presented (de Lafuente and Ruiz, 2004). This raises the question whether illusory bias and uncertainty changes in classifying Müller-Lyer images that are presented diagonally, rotated by a number degrees to the left or to the right. Simple cells in HMAX consist of linear oriented filters, and are present in multiple orientations. The max pooling operations combine input from these and provide an output that is invariant to rotation. As a result, we would predict no difference in results when processing versions of the Müller-Lyer illusion in HMAX rotated at any arbitrary angle. This prediction is also consistent with human studies. While a number of illusions demonstrate an increase in magnitude when presented in a tilted condition, there is no difference in magnitude for the MLI (Prinzmetal and Beck, 2001).

In our last study, we recruited a previous version of HMAX known as FHLib, a Multi-scale Feature Hierarchy Library (Mutch and Lowe, 2008). In the current study, a more recent, GPU-based version of HMAX, known as CNS: Cortical Network Simulator (Mutch et al., 2010) was used. The main difference between these architectures was a linear classifier replacing the SVM in the final layer of the more recent code. The network setup between architectures was identical: one image layer followed by four layers of alternating S and C layers. Both had the same levels of inhibition (50% of cells in S1 and C1). The image layer contained 10 scales, each level 2^1/4^ smaller than the previous. Compared to our previous study, we were able to replicate similar levels of bias despite a change in the classifier, demonstrating that our result is robust and dependent upon properties of the HMAX hierarchical architecture, rather than the small differences between the implementation of these two related models.

Reflecting upon the implication of our results for other models, we would predict that those that have a similar hierarchical architecture would exhibit similar trends. That is, comparable networks would demonstrate increased bias with decreased precision when categorizing MLI images with less variance. Considering models that only contain filtering operations (akin to layers of simple cells) we would observe an illusory effect that may also be exacerbated compared to those with more complex operations, with low accuracy and precision. Examples of would include the model of Bertulis and Bulatov (2001).

The reduction of bias in computer vision systems has significant ramifications for applications such as automated driving, flight control and landing, target detection and camera surveillance. Correct judgment of distances and object dimensions in these systems could affect target accuracy and reduce the potential for crashes and errors. Our hypothesis that increasing positional variance in the stimuli would reduce the magnitude of illusory bias could be extended to include other forms of variance, such as image rotation, articulation or deformation, hence examining the generality of this proposal. Furthermore, it would be informative to test the generality of the results presented in this study in other computational models. If a general effect could be confirmed, then we would advise the implementation of many forms of input variance during training to improve their judgment capabilities, providing more accurate and precise information.

Our work not only has implications for the field of computer science, but also for psychology. Computational models allow manipulations of parameters that are impossible or impracticable to perform in human subjects, such as isolating the contributions of different neural structures to the effect. Artificial architectures allow us to make predictions about overall human performance as well as how performance changes from layer to layer within the visual system. Considering that this model not only provides an overall system performance (C2 output), but also supplies information at multiple levels of the architecture that correspond approximately to identifiable neural substrates, it may be possible to test the model's predictions with neuroimaging data. Using functional magnetic resonance imaging (fMRI), we could obtain blood-oxygen-level dependent (BOLD) signals at different levels of the visual cortices of observers viewing the MLI compared to a control condition (using a similar method to that described by Weidner and Fink, 2007). Then by applying a classifier to these signals, we could map this information to changes in model bias and quantify how well the model matches human brain data. This forms a possible direction for future research.

## Funding

Astrid Zeman is supported by a CSIRO Top-Up Scholarship and the Australian Postgraduate Award (APA) provided by the Australian Federal Government. Astrid Zeman is also supported by the Australian Research Council Centre of Excellence for Cognition and its Disorders (CE110001021) http://www.ccd.edu.au.

### Conflict of interest statement

The authors declare that the research was conducted in the absence of any commercial or financial relationships that could be construed as a potential conflict of interest.
